# Evolution insolite d'une plaie complexe de la voie biliaire principale post cholécystectomie cœlioscopique

**DOI:** 10.11604/pamj.2016.23.150.6882

**Published:** 2016-03-31

**Authors:** Hedfi Mohamed, Bouhafa Hala, Elcadhi Youssef, Cherif Abdelhedi, Sassi Karim, Sridi Azza, Chouchene Adnen

**Affiliations:** 1 Service de Chirurgie Hôpital des FSI La Marsa, 2 rue Fadhel Ben Achour, La marsa 2070, Tunisie

**Keywords:** Plaies, voies biliaires, coelioscopie, Injuries, bile duct

## Abstract

Depuis l'avènement de la chirurgie coelioscopique de la lithiase biliaire le nombre de plaies des voies biliaires a sensiblement augmenté dans la littérature en rapport avec la courbe d'apprentissage des opérateurs. Les plaies méconnues peuvent avoir des conséquences immédiates dramatiques et évoluer vers la péritonite biliaire. Ailleurs la réparation des fistules biliaires externes au stade de dilatation des voies biliaires nécessite une anastomose bilio digestive ou des résections hépatiques réglées.

## Abstract (en)

Since the advent of laparoscopic surgery of cholelithiasis the incidence rate of bile duct injuries has increased significantly in the literature in relation to the operators’ learning curve. Unknown injuries can have dramatic, immediate consequences and progress to bile peritonitis. Moreover surgical repair of external biliary fistula at the stage of bile duct dilatation requires biliodigestive anastomosis or liver resections “réglées”.

## Introduction

L'incidence des plaies iatrogènes de la voie biliaire principale a augmenté depuis l'avènement de la cholécystectomie laparoscopique [1]. Ces lésions sont méconnues dans plus de la moitié des cas au cours de l′intervention coelioscopique initiale. Par ailleurs leur diagnostic tardif plusieurs jours après l′intervention entraine une mortalité et une morbidité significativement accrues et des implications medico légales [2]. La courbe d'apprentissage de l’équipe chirurgicale initialement identifiée comme cause principale de l'augmentation de la fréquence des plaies biliaires, ne peut à elle seule expliquer cette complication. Nous proposons un cas clinique typique de méconnaissance d'une plaie biliaire complexe et nous discutons les différentes étapes de son évolution à la lumière des publications récentes.

## Patient et observation

**Figure 1:**
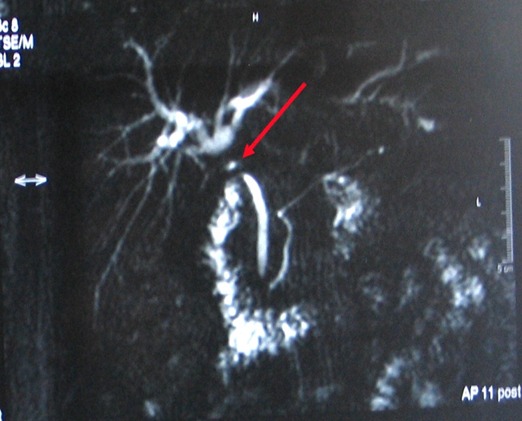
BILI-IRM montrant une dilatation modérée des voies biliaires intra hépatiques avec une perte de substance allant de la plaque hilaire jusqu'au bord supérieur du duodenum

**Figure 2:**
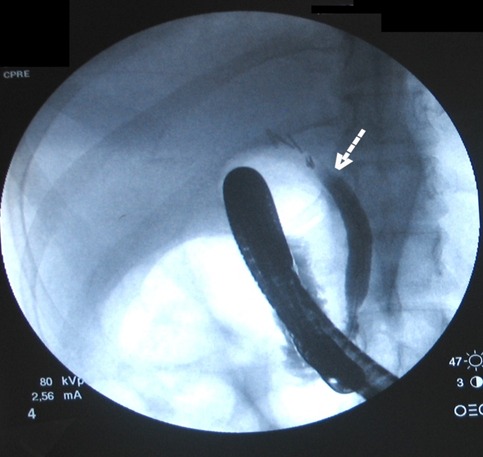
Section complète de la voie biliaire principale avec une fistule bilio bulbaire fonctionnelle entre la plaque hilaire et le duodénum

Mme B.L âgée de 36 ans sans, a été opérée par voie coelioscopique en Février 2006 pour lithiase vésiculaire simple; les suites opératoires étaient marquées par l'apparition d'un sub ictère, au 7e jour post opératoire, un traitement symptomatique lui a été prescrit sans explorations. Devant l'aggravation de son état général la patiente a consulté à nos urgences au 16ème jour post opératoire d'où son admission. L'examen avait trouvé une patiente dont l’état général est altéré sans choc septique, apyrétique, sub ictérique,avec une matité des 2 flancs et un bombement indolore du cul de sac de Douglas au toucher rectal. la biologie avait montré une cholestase: hyperbilirubinemie à prédominance conjuguée (70/48 µmole /l), phosphatases alcalines à 435UI/l, GGT à 137UI/l, un TP bas à 39% et des transaminases normales. L’échographie ainsi que le scanner abdominal ont révélé un épanchement intra-péritonéal de grandes abondances diffuses dont la ponction a ramené un liquide biliaire. L'indication d'une laparotomie a été posée avec le diagnostic de plaie biliaire L'exploration chirurgicale avait trouvé un épanchement intra péritonéal bilieux d'environ 3 litres en rapport avec une perte de substance de la voie biliaire principale (VBP) s’étendant de la convergence des canaux hépatiques droit et gauche jusqu'au bord supérieur du duodénum;il a été réalisé une toilette péritonéale avec drainage sélectif des canaux hépatiques biliaires droit et gauche. L’évolution immédiate etait favorable et les drains ont donné 2litres de bile par jour puis se sont progressivement taris vers le 20e jour post opératoire. L'echographie de contrôle avait montré une dilatation modérée des canaux hépatiques droit et gauche sans épanchement intra péritonéal. Une cholangiographie IRM a été demandé au 4e mois de l’évolution, cette dernière avait conclu à une image de defect biliaire de 18 mm s’étendant de la plaque hilaire jusqu'au bord supérieur du duodénum associée à une dilatation modéré des canaux hépatiques droit et gauche à 7 mm (Figure 1). La CPRE a conclu à une section complète de la VBP avec une fistule bilio bulbaire fonctionnelle entre la plaque hilaire et le duodénum expliquant l'absence de dilatation importante des voies biliaires et le tarissement de l’écoulement du drain (Figure 2). A 8 mois d’évolution la patiente a développé un ictère flamboyant en rapport avec une sténose de la fistule bilio digestive, mais sans angiocholite aigue grave. La patiente a été réopérée: il a été réalisé une déconnection de la fistule bilio digestive et une anastomose hepatico jéjunale sur une anse montée en Y. L’évolution était favorable.

## Discussion

Avec un meilleur apprentissage et contrôle de l'approche laparoscopique; l'incidence des plaies biliaires a nettement diminué pour revenir à des chiffres proches de la laparotomie. Nuzzo rapporte dans la monographie nationale de la société Italienne de chirurgie, publiée en 2002 que sur 56591 cholécystectomies laparoscopiques réalisées dans 184 unités de chirurgie générale pendant une période de trois ans (1998-2000), 235 plaies biliaires ont été observées, soit une prévalence de 0,41%. Dans 178 cas (76%) il s'agissait de lésions « majeures », selon la classification de Bismuth et dans 57 cas (24%) des lésions « mineures ». L'incidence des lésions biliaires majeures a donc été de 0,31%. L’âge moyen des plaies biliaires en coelioscopie est de 43 ans et majoritairement féminin dans la littérature [1–5]. Le mécanisme lésionnel de ces plaies est dans la majorité des cas lié à la confusion entre la voie biliaire extra hépatique et le canal cystique favorisée par une traction excessive sur l'infundibulum vésiculaire survenant dans environ 40% des cas [6]. La dissection d'un collet vésiculaire adhérent, lorsque le pédicule hépatique est inflammatoire risque d′entraîner une blessure du canal hépatique commun ou du canal droit dans les cholécystectomies difficiles [1]. D'autres mécanismes sont incriminés: - Débordement d'un clip mordant sur la VBP qui risque de se nécroser ou de se sténoser; - Le mauvais usage de l′électrocoagulation [7] qui risque, par contact direct de la VBP, ou même par courant induit, d′entraîner une nécrose de la paroi biliaire, suivie de perforation, ou même d'une sténose canalaire longue d′apparition secondaire. - Un canal cystique très court facilite la confusion entre cystique et cholédoque. - La présence d′anomalies anatomiques des voies biliaires, en cas de convergence étagée, est souvent incriminée comme l'abouchement du canal cystique dans un canal sectoriel ou segmentaire. De même des canaux biliaires aberrants, peuvent s′implanter dans la vésicule (canal de Luchka) ou le canal cystique. Ces anomalies biliaires sont en fait rares (2 à 12% selon les séries) et n′expliquent qu′un faible pourcentage des traumatismes de la VBP au cours de la cholécystectomie. En outre, les plaies sont plus fréquemment associées aux cholécystites aigues qu'aux lithiases simples. Il est par conséquent important de savoir renoncer à la voie coelioscopique et se convertir en laparotomie en cas de difficulté dans l′identification du canal cystique ou de la VBP, d′écoulement biliaire inexpliqué, et en cas d′hémorragie difficilement contrôlable. Le rôle de la Cholangiographie per opératoire faite de façon systématique, est très controversé du fait qu'elle ne prévient pas les plaies car celles-ci sont souvent faites après la cholangiographie [8, 9]. Elle peut être toutefois très utile lors de l'isolement du cystique si l'on a tendance à le confondre avec la VBP. Dans ces cas, la cholangiographie permet la reconnaissance de cette erreur et évite de transformer une plaie latérale de la voie biliaire principale, réparable immédiatement et en général sans conséquence, en une section complète ou en l'exérèse d'un segment biliaire entier. le rôle préventif de la cholangiographie per opératoire systématique n'a pas été démontré [10]. Toutefois, en cas de suspicion d'une plaie biliaire au cours d'une cholécystectomie Il est indispensable de disposer en permanence de l'appareillage adapté, d'avoir une habitude suffisante de sa réalisation et une bonne connaissance de l'anatomie radiologique des voies biliaires.

Les plaies biliaires survenues en coelioscopie sont plus graves qu'en laparotomie, en effet lors d'une étude rétrospective par Chaudhary et al [2] comparant les stades des plaies biliaires en laparotomie à la laparoscopie, 63% des plaies biliaires survenues en laparoscopie étaient classées Bismuth III-IV, contre seulement 32% en laparotomie. Le bilan lésionnel préconisé avant réparation est un temps très important et un diagnostic pré opératoire incomplet peut être à l'origine d'un échec du traitement chirurgical [2, 4]. Les examens complémentaires réalisés sont le plus souvent une échographie, une bili- IRM [1] et parfois une cholangiographie par voie rétrograde. Le traitement endoscopique constitue une alternative séduisante dans la prise en charge des plaies de la VBP. Liguory et Lefebvre [1] ont proposé une sphinctérotomie endoscopique et une intubation par un drain naso biliaire ou d′une prothèse qui permet d'exclure la plaie et lui permettre de cicatriser. La durée du drainage varie de 2 à 12 mois. Les indications du traitement endoscopique paraissent en réalité très restreintes essentiellement pour les petites plaies latérales de la VBP découvertes en post opératoire immédiat. Dans certains cas la cicatrisation aboutit à une sténose et le drain est insuffisant pour la calibrer Le traitement chirurgical est donc le traitement de choix. Il consiste une anastomose bilio digestive sur une anse montée en Y. La confection de l′anse en Y est sans particularité, mais le temps biliaire doit être exécuté avec une grande rigueur [8, 9]. L'anastomose biliojéjunale latérolatérale doit être aussi large que possible sur une voie biliaire saine avec un affrontement mucomuqueux et sans tension, ce qui peut nécessiter une dissection hilaire et la résection d'une partie de la voie biliaire principale. Le meilleur moment de la réalisation d'une réparation définitive ne fait pas l'objet d'un consensus. En cas d′ictère lié à une obturation complète de la VBP, après le tarissement d'une fistule biliaire externe la dilatation de la voie biliaire atteint en 3 à 4 semaines un calibre suffisant pour la réalisation d′une bonne anastomose [7]. Pour d'autres auteurs [5] il faut attendre 8 à 12 semaines pour obtenir une régression des phénomènes inflammatoires et une dilatation des voies biliaires après le tarissement d'une éventuelle fistule biliaire. Chapman et al [10] critique une telle attente car elle serait responsable d'une morbidité plus importante il opte donc pour une réparation plus précoce à 4 semaines. Au cours de cette attente, on peut observer une diminution paradoxale de l′ictère qui pourrait faire croire à une guérison. Cette rémission est liée en fait à l′apparition d′une fistule biliaire interne par ouverture du cul-de-sac biliaire dans le duodénum,comme c'est le cas dans notre observation. La survenue de cette communication bilio duodénale, souvent étroite et tortueuse, ne remet pas en cause l′indication de la réparation biliaire. Les échographies successives permettent de suivre le calibre de la voie biliaire hilaire et des voies biliaires intra hépatiques, et de choisir le moment opportun pour la réparation. Seule la survenue de poussées d′angiocholite répétées pourrait faire avancer la date de cette réparation.

## Conclusion

Les plaies de la voie biliaire principale au cours de la cholécystectomie laparoscopique sont un accident aux conséquences graves pour le patient et pour le chirurgien. La fréquence réelle semble être encore sous-estimée. Il est nécessaire pour le chirurgien d’être prudent et vigilant pour les éviter ou à défaut de les reconnaître en per opératoire pour les réparer immédiatement. Le rôle de la cholangiographie per opératoire systématique est encore critiqué car elle ne prévient pas les plaies mais reste un argument juridique lors des expertises. La réparation consiste en la réalisation d'une anastomose bilio-digestive ou des résections hépatiques réglées.

## References

[1] Nuzzo G, Giuliante F, Persiani R (2004). Le risque de plaies biliaires au cours de la cholécystectomie par laparoscopie. J Chir (Paris).

[2] Z'graggen K, Wehrli H, Metzger A, Buehler M, Frei E, Klaiber C (1998). Complications of laparoscopic cholecystectomy in Switzerland: à prospective 3-year study of 10,174 patients, Swiss Association of Laparoscopic and Thoracoscopic Surgery. Surg Endosc..

[3] Wudel LJ, Wright JK, Pinson CW, Herline A, Debelak J, Seidel S (2001). Bile duct injury following laparoscopic cholecystectomy: a cause for continued concern. Am Surg..

[4] Slater K, Strong RW, Wall DR, Lynch SV (2002). Iatrogenic bile duct injury: the scourge of laparoscopic cholecystectomy. ANZ J Surg..

[5] Bismuth H, Lazorthes F (1981). 83rd Congress of the French Surgical Society (Paris, 21-24 September 1981), Second report: Operative injuries of the common biliary duct. J Chir (Paris)..

[6] Carroll BJ, Birth M, Phillips EH (1998). Common bile duct injuries during laparoscopic cholecystectomy that result in litigation. Surg Endosc..

[7] Davidoff AM, Pappas TN, Murray EA, Hilleren DJ, Johnson RD, Baker ME (1992). Mechanisms of major biliary injury during laparoscopic cholecystectomy. Ann Surg..

[8] Borie F, Millat B (2003). Cholangiographie peropératoire par laparoscopie: Comment et pourquoi le faire?. J Chir (Paris).

[9] Vons C (2003). Une cholangiographie systématique au cours d'une cholécystectomie par laparoscopie est-elle vraiment justifiée?. J Chir (Paris).

[10] Phillips EH (1993). Routine versus selective intraoperative cholangiography. Am J Surg..

